# Semiochemical signatures associated with differential attraction of *Anopheles gambiae* to human feet

**DOI:** 10.1371/journal.pone.0260149

**Published:** 2021-12-03

**Authors:** Maurice O. Omolo, Isaiah O. Ndiege, Ahmed Hassanali

**Affiliations:** 1 Department of Chemistry, School of Pure and Applied Sciences, Kenyatta University, Nairobi, Kenya; 2 Behavioural and Chemical Ecology Department (BCED), International Center of Insect Physiology and Ecology (ICIPE), Nairobi, Kenya; 3 Department of Pure & Applied Chemistry, Faculty of Science, Masinde Muliro University of Science and Technology (MMUST), Kakamega, Kenya; 4 Center for African Medicinal & Nutritional Flora & Fauna (CAMNFF), Masinde Muliro University of Science and Technology (MMUST), Kakamega, Kenya; INRA-UPMC, FRANCE

## Abstract

**Background:**

Several human-produced volatiles have been reported to mediate the host-seeking process under laboratory conditions, yet no effective lure or repellent has been developed for field application. Previously, we found a gradation of the attractiveness of foot odors of different malaria free individuals to *Anopheles gambiae sensu stricto* Giles. In this study, foot odor of the individual with the most attractive ‘smelly’ feet to the *An*. *gambiae* was collected, analyzed and attractive blend components identified.

**Methods:**

The foot odor of the individual with the most attractive ‘smelly’ feet to the *An*. *gambiae* was trapped on Porapak Q and analyzed by gas chromatography-linked mass spectrometry (GC-MS). Specific constituents perceived by the insect olfactory system were then identified by GC-linked to electro-antennography detector (GC-EAD) and characterized by GC-MS. The contribution of each constituent to the behavioral response of *An*. *gambiae* was assessed through subtractive assays under semi-field conditions in a screen-house using Counter Flow Geometry (CFG traps) baited with (i) the blend of all the EAD-active and (ii) other blends containing all components with exclusion of one component at a time. The number of mosquitoes trapped in the baited CFG traps were compared with those in the control traps.

**Results:**

Eleven major and minor constituents: 2 carboxylic acids, six aldehydes, two ketones and one phenolic compound, were confirmed to be EAD-active. The contribution of each constituent to the behavioral response of *An*. *gambiae* was assessed through subtractive assays under semi- field conditions. Exclusion/ subtraction of one of the following compounds: *i-*butyric acid, *i-*valeric acid, *n*-octanal, *n*-nonanal, *n*-decanal, *n*-dodecanal, undecanal or *n*-tridecanal, from each blend led to reduction in the attractiveness of all the resulting blends, suggesting that all of them are critical/important for the attractiveness of the foot odor to *An*. *gambiae* mosquitoes. However, exclusion/subtraction of 4-ethoxyacetophenone, 4-ethylacetophenone and/or 2-methylphenol, led to significant enhancements in the attractiveness of the resulting blends, suggesting that each of these compounds had repellent effect on *An*. *gambiae ss*. Undecanal exhibited kairomonal activity at low natural concentrations under semi-field conditions but repellent activity at high unnatural conditions in the laboratory. Furthermore, the comparison of the mean mosquito catches in traps baited with the nine-component blend without 4-ethoxyacetophenone, 4-ethylacetophenone and the complete foot odor collection revealed that the former is significantly more attractive and confirmed the repellent effect of the two carbonyl compounds at low natural concentration levels.

**Conclusion:**

These results suggest that differential attractiveness of *An*. *gambiae* to human feet is due to qualitative and/or qualitative differences in the chemical compositions of the foot odors from individual human beings and relative proportions of the two chemical signatures (attractants versus repellents) as observed from the ratios of the bioactive components in the foot odors of the most attractive and least attractive individuals. Chemical signature means the ensemble of the compounds released by the organism in a specific physiological state. The chemical signature is emitter-dependent, but does not depend on receiver response. Thus, there is only one chemical signature for one individual or species that may eventually include inactive, attractive and repellent components for another organism. The nine-component attractive blend has a potential as an effective field bait for trapping of malaria vectors in human dwellings.

## Introduction

Female *Anopheles gambiae sensu stricto* Giles (Diptera: Culicidae) is one of the most important vectors of malaria in sub- Saharan Africa. It is highly anthropophilic with majority of the females being endophilic after blood meals, [1–3]. Consequently, the proportion of the vector population infected with *Plasmodium falciparum* sporozoites is generally much higher for *An*. *gambiae s*.*s*. than any other *Anopheles* species [4]. Generally, female mosquitoes searching for blood meals make specific selection of the hosts based on olfactory cues and body temperature among other factors [5, 6]. Mosquito species with distinct host preferences respond differently to human or cattle odors [7–10], and single odor components [11–13]. For instance, host location by *An*. *gambiae s*.*s*. is mediated by the volatile odors from human body and breath [14–16]. The human breath, contains semio-chemicals that elicit behavioral responses in *An*. *gambiae* both as kairomones and allomones while the differences in attractiveness of various people to *An*. *gambiae* depend on the variations in their skin micro-biota [17, 18] and repellent volatiles [14]. The exhaled CO_2_ is generally a kairomone for all mosquitoes [19], even though, it is less attractive to the anthropophilic *An*. *gambiae* than it is to the zoophilic and opportunistic *An*. *arabiensis* [10, 20, 21]. The attractiveness of human beings to *An*. *gambiae s*.*s*. [14, 22] and *Aedes aegypti* [23] differ from one individual to the other due to the variations in human body odors [14, 22, 24–27], which depends on the population and type of the skin microbes resident on the body [16–18, 25].

Host-attractant baited traps and targets are some of the promising environmentally friendly technologies currently being considered for mosquito control since the beginning of the last decade [15, 28, 29]. Volatile attractive host odor has been recognized to play an important role in guiding malaria vectors to the human host and preferred feeding site(s) [15, 16, 24, 30–34]. Studies on the selection of biting sites based on blood-meal analysis have revealed that *An*. *gambiae s*.*s*. has a preference for feeding on the feet more than any other part of the human body [32, 33]. The odor from human feet is more attractive to *An*. *gambiae s*.*s*. than from cow leg [35]; and washing of the feet substantially reduces their attractiveness to *An*. *gambiae* [24]. It has also been reported that placing worn socks next to a blood-feeding membrane device enhances landing, feeding and fecundity in *An*. *gambiae* and *An*. *stephensi* [36], which may be attributed to foot odor generated by microbes [37]. It has further been established that *An*. *gambiae* is attracted to a synthetic mixture of fatty acids from human sweat [38] and Limburger cheese (used to mimic human foot odor) [39, 40]. The fatty acids are products of the human sebum from microbial breakdown; and are responsible for the distinctive human olfactory signature [17, 41]. The observations strongly suggest that there are site-specific chemical cues that guide the anthropophilic female *An*. *gambiae* mosquitoes in host location and feeding site selection, which has been attributed to site-specific semio-chemicals [42].

The human odor constituents that have previously been identified or implicated as candidate attractants include: carbon dioxide, *L*-lactic acid, 1-octen-3-ol, acetone, ammonia, and several aldehydes, alcohols, ketones and carboxylic acids [1, 11, 43–45]. However, none of these: including carbon dioxide with or without some of the above mentioned constituents, individually match the attractiveness of human odor under field conditions [43, 46]. Identification of the host-specific volatile semio-chemicals involved in selection of the human feet as a preferential feeding site by *An*. *gambiae* and their subsequent exploitation in host-odor baited traps and targets would therefore help to reduce the threat and intensity of transmission of malaria by *An*. *gambiae* especially when deployed jointly with entomo-pathogens in the control of wild mosquito populations. Furthermore, it would help in sampling and monitoring of natural mosquito populations, forecasting disease outbreaks, deciding the time/period for insecticide application and anti-malarial drugs procurement. We herein report the identification of semio-chemicals (kairomones and repellents) from the human foot odor and their blend(s) that are responsible for the observed preference for the human feet as a feeding site for female *An*. *gambiae*; demonstrate their importance as mosquito attractants and discuss their potential in the sampling, monitoring and control of the malaria vector populations under semi-field conditions.

## Materials and methods

### Preparation of the adsorbent filter

Porapak Q (80–100 mesh, Germany), was used as the adsorbent for the sampling of the feet odors [22]. Filter papers (Whatman No. 1) were used to make the pockets for packing the adsorbents. The papers were first cut into small strips (10 x 3.0 cm), folded into two equal halves (5 x 3.0 cm) and then along the sides to form pockets (5 x 2 cm). One short side and the two long ones were sewn using white cotton thread on a sewing machine to form the adsorbent pockets/containers. Similar pockets were made using cream wire mesh sheets (Estal Mono, 120T mesh; Swiss Silk Company, Switzerland) with the three folded edges stapled.

The paper pockets were packed with 500 mg of Porapak Q powder, the open end folded twice and stapled. The filled paper pockets were enclosed in the cream mesh sheets to protect them from getting torn. The pockets containing the adsorbents were cleaned in a Soxhlet extractor using dichloromethane for 72 h, removed using a pair of forceps and dried in an oven for 2 days at 40°C. Porapak Q was activated under an inert atmosphere of N_2_ gas in a GC oven at 160°C for 5 h [47].

White cotton and nylon socks were used for bulk adsorption of the foot odors. The socks were bought from the same batch, washed well with bar soap, rinsed and air dried before being worn by the human subjects.

### Collection of the foot odors

Human subjects were graded in order of the attractiveness of their feet odor to *An*. *gambiae s*.*s*. [22]. Collection of foot odor was carried out overnight from the feet of the most and least attractive subjects, in two huts (3.2 x 2.7 x 2.4 m) within a screen-house (11.5 x 7.1 x 3 m) at ICIPE Mbita Point Field Research Station (Thomas Odhiambo Campus) on the southern shores of Winam Gulf of Lake Victoria in Nyanza Province, Kenya (00° 25’ S, 34° 13’ E). At about 8.30 pm (EAT) or 5.30 pm (GMT), Soxhlet-cleaned (dichloromethane) cotton thread was used to tie the sachets of adsorbents around the phalanges of the feet of the human subjects (S1 Plate). The subjects were then assisted to wear the cotton/ nylon socks, which they slept in for 10–12 hours. The sachets were removed in the morning (7.00 am, EAT) of the following day, packed in glass bottles (100 mL) with quick fit stoppers and stored in a cool box from where they were transferred into a freezer (– 20°C) until the desorption of the volatiles was carried out.

### Extraction of volatiles from the adsorbents

The adsorbent materials in the pockets were carefully transferred into Pasteur pipettes, equipped with clean glass wool, eluted with 2 mL dichloromethane (99.9% HPLC grade, Aldrich Chemical Co., UK) and the eluent collected in clean vials (4 mL) under ice salt mixture (-4°C) and stored in the freezer until when required for bioassay and chromatographic analysis [47, 48].

### Analysis of volatiles

The gas chromatographic separation was performed on a Hewlett Packard (HP) model 5890 Series II capillary gas chromatograph, equipped with a split-less injector system, a flame ionization detector (FID) coupled to an integrator (HP 3393A Series II). Cross-linked methylsilicone, 50 m x 0.2 mm x 0.33 μm Hewlett Packard capillary column was used; with white-spot nitrogen, at a flow rate of 0.7 ml/min, as the carrier gas; and hydrogen (analytical grade) together with medical air (pure oxygen) as fuel. The column temperature was initially set at 40°C (5 min), increased to 200°C @2°C/min (15 min) and finally to 285°C @ 3°C/min (75 min). The dichloromethane-eluted odor was cooled in ice, and concentrated to 100 μL (using a gentle stream of nitrogen), and aliquots (2 μL) of the concentrate injected into the GC or GC-MS. GC–MS analyses were carried out on a HP 8060 Series II gas chromatograph coupled to a VG Platform II mass spectrometer. The mass spectrometer was operated in the electron ionization (EI) mode at 70 eV and an emission current of 200. _A. The temperature of the source was held at 180°C and the multiplier voltage was 300 V. The pressure of the ion source was held at 9.4 × 10^−6^ mBar, while that of the analyzer (MS detector) was 1.4 × 10^−5^ mBar. The spectrometer had a scan cycle of 1.5 s (scan duration of 1 s and interscan delay of 0.5 s). The mass range was set at m/z 1–1400. The scan range for the samples was however from m/z 38–650. The instrument was calibrated using heptacosafluorotributylamine [CF3(CF2)3]3N (Apollo Scientific Ltd. UK). The GC column used was the same as the one described for the GC analysis except for the film thickness of 0.5 μm. The temperature program was the same as that for the GC analysis Omolo et al. [22].

### Mosquitoes for bioassays

*An*. *gambiae s*.*s*. mosquitoes used in the experiments were unfed and unmated females from a colony reared at ambient temperature (26–28°C) and humidity (70–80% R.H.), at the International Centre for Insect Physiology and Ecology (ICIPE) Mbita Point Field Research Station (Thomas Odhiambo Campus) on the southern shores of Winam Gulf of Lake Victoria in Nyanza Province, Kenya (00° 25’ S, 34° 13’ E). Rearing temperature and relative humidity in the adult insectaria were 26–28°C and 70–80%, respectively. Adults were maintained on 6% glucose and female mosquitoes were routinely offered human arm to feed upon. The larvae were reared in plastic basins (50 cm diameter and 16 cm height) at a density of 200–250 larvae per basin in 3 L of fresh water from Lake Victoria at 32–36°C and fed on Tetramin^®^ fish food (Tetra GmbH, Germany). Pupae were collected daily and kept in mesh-covered cages (30 cm × 30 cm × 30 cm) containing 6% glucose solution in filter paper wicks, which served as their food upon emerging into adults. Adult females were used for the experiments when 5–8 days old and with no prior access to a blood meal, as previously described by Omolo et al. [22].

### GC-EAD analysis and bioassay of human foot odor

Antennae of 3–4 day-old female *An*. *gambiae* s.s. mosquitoes were used for the coupled gas chromatography-electroantennographic detector (GC-EAD) analysis [1]. GC-EAD analysis was done according to the procedure established by Ephrussi [49]. Briefly, a glass micro-pipette containing Beadle-Ephrussi saline [49] was inserted through the inter-segmental membrane between the abdomen and the thorax of the insect. The micro-pipette’s fine tip was pushed through the thorax, then the neck and into the head. The other end of the micro-pipette was sheathed over a silver wire, the recording electrode, which was connected to the input of a universal AC/DC UN-05 amplifier (Syntech, Netherlands). To complete the circuit, the distal end of the antenna was nipped off with a scalpel and the open end inserted into a similar glass micropipette containing the saline and also sheathed over a silver wire electrode that was grounded. GC-EAD tests were performed on a Hewlett-Packard (HP) 5890 Series II capillary gas chromatograph equipped with an effluent splitter, flame ionization detector (FID) and HP Ultra 1 (cross-linked methylsilicone gum) column (50 m × 0.2 mm × 0.33 μm) using white-spot nitrogen as the carrier gas at a flow rate of 0.80 mL/min. The oven temperature was initially maintained at 60°C for 5 minutes following injection of the sample, increased to 280°C (@ 5°C/min), and held there for 15 minutes. The effluent from the capillary column was split in ratio of 1:1 into two 50 cm long deactivated silica columns: one connected to the FID, and the other to a 5 mm (i.d.) stainless steel tube that was focused onto the antennal preparation. Makeup gas was introduced just before the split point at a flow rate of 40 mL/min to accelerate the effluent through the deactivated silica columns. The deactivated transfer line carrying the effluent over to the antennal preparation was maintained at 150 °C by a temperature control unit (THC-3, Syntech, Netherlands). Aliquots (6–8 μL) of the concentrated human foot odor extracts were injected into GC-EAD equipment and both EAD and GC signals monitored synchronously using a program on a GC-EAD interface card (Syntech, Netherlands) installed in a PC (Harvard Professional Computer, American Megatrends Inc.).

### Preparation of the EAD active components for field bioassay

The amounts of the EAD-active components tested individually or in blends were determined as follows: 0.2 μL (0.1714 mg) of *p*-cymene (99% GC grade, density 0.857 g/mL, Aldrich Chemicals Co., UK) was added to 2 mL (2000 μL) of the foot odor extract in dichloromethane (99.9% HPLC grade, Aldrich Chemical Co., UK) to provide a solution containing 0.0001 μL (0.0000857 mg) of the internal standard (*p*-cymene) in 1 μL of the mixture, which was injected (2 μL) into the GC column for analysis. GC analysis was done as described above [22]. The relative peak area (0.678) of the internal standard was correlated to the amount of the compound (0.0002 μL or 0.0001714 mg) in the 2 μL mixture injected into the GC column. The result obtained was then used to calculate the amount of each of the EAD active compounds present in the 2 μL of the foot odor extract by multiplying relative area of each of the EAD-active components by the factor 0.0002528 mg (0.0001714/0.678) and consequently the amount of each component in 1 mL of the foot odor extract (concentration in g/mL) determined. The concentration of EAD active component was then scaled up by a factor of 50 to obtain measurable amounts of the respective components and blends required to prepare 50 mL of the test solution of same concentrations as the foot odor extract in acetone (99.9% HPLC grade, Aldrich Chemical Co., UK). The ratios of the EAD-active components in the blend were determined from the respective amounts used to prepare the 50 mL stock solutions.

### Semi field subtraction assay of the EAD active chemical components of the foot odor

All the 11 EAD-active components of the human foot odor were subjected to subtraction bioassays in form of blends under semi-field conditions. The first blend had all the 11 EAD-active components, while the rest of the blends had a total of 10 components, with each blend missing one particular component at a time, which would be present in the rest. A total of 12 blends were prepared in acetone for the semi-field assays in 8 replicates (between 8 p.m. and 6 a.m. for 8 nights). Control experiments contained 99.9% HPLC grade acetone only. Three additional blends were similarly prepared in acetone as earlier described but with more than one EAD-active components of the human foot odor missing, and assayed.

**Fig 1:**
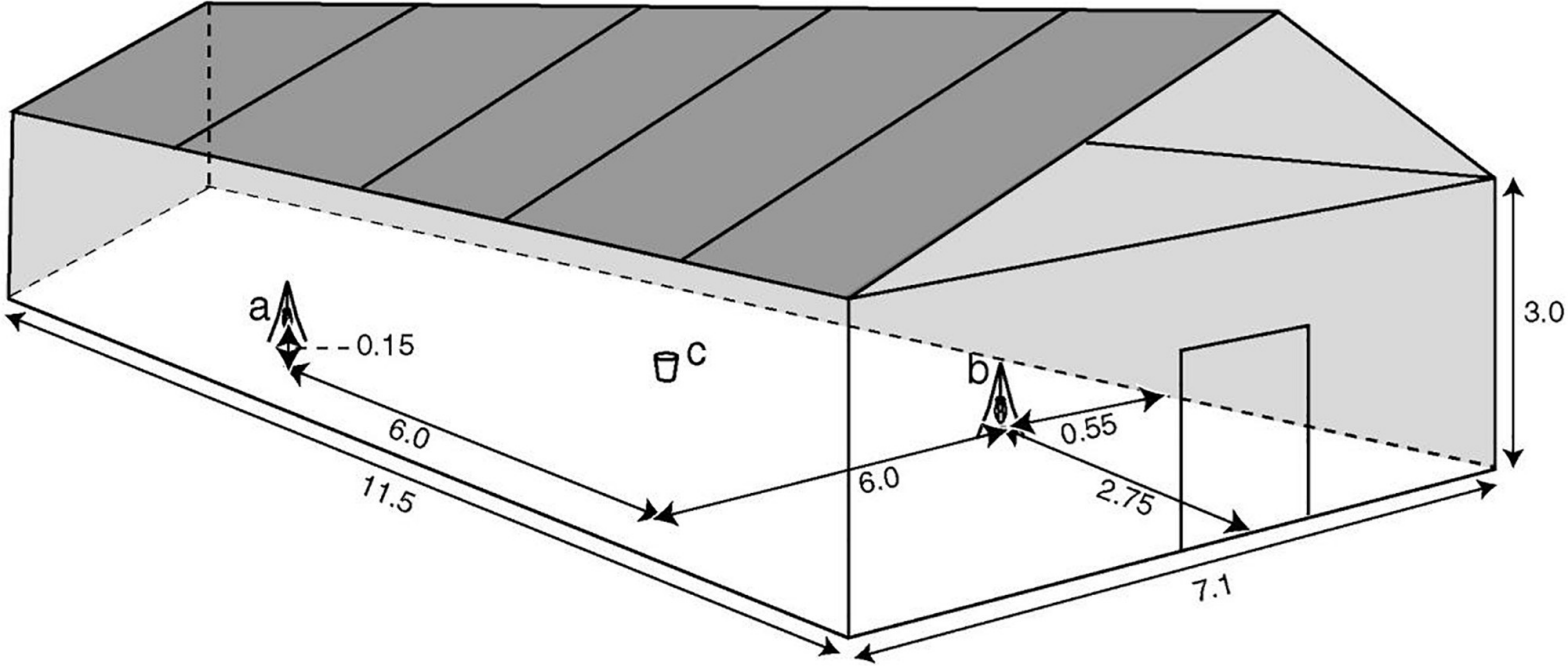
Diagonal arrangement of test and control CFG traps and mosquito release point. a and b represent the CFG traps at 2 different locations with the cup (c) at equidistant point from each of the traps in the screen house.

A 5 mL syringe was used to apply 3 mL of a blend solution evenly onto cleaned cotton socks, which was then used as the bait in a CFG trap after the socks had been left in the open for 30 min. to let the acetone (solvent) to evaporate. The same amount (3 mL) of acetone was dispensed on another set of cleaned socks and also given 30 min. to allow for evaporation before being put in another CFG trap to serve as the control. The test and control CFG traps were arranged diagonally in the screen house and 200 starved female *An*. *gambiae* mosquitoes (3–5 days old) released from a cup placed at a central point (Fig 1) The test and control CFG traps were interchanged between the two sites (A and B) after every two successive nights such that, 4 replicates were done at each of the sites A and B for both the test and control CFG traps. The experiment was replicated 8 times for each of the blends of EAD-active components. The catch size due to the blend effect of each of the 12 blends was computed using the equation: N = T—C where by N = catch size due to a blend, T = number of mosquitoes caught in test trap, and C = number of mosquitoes caught in control trap [50]. The transformed mosquito catch size data was subjected to statistical analysis of the means using Student-Newman-Keuls (SNK) test as previously described in our earlier publication [22].

### Comparison of the attractive blend of the human foot odor with other known devices

The kairomonal activity of the eight-component blend of human foot odor was further demonstrated in dual choice assays where mosquito catch sizes from CFG traps baited with the blend of the 8 attractive constituents were compared to those from: (a) CDC USA light traps in a screen-house; (b) human-baited bed-net trap [28]; and (c) CFG traps with socks baited with natural foot odor blend from the most attractive volunteer in a bedroom of a household with the owner (volunteer) sleeping on his bed.

### Mosquito repellency assay of the repellent blend constituents of the human foot odor

Laboratory-reared female *An*. *gambiae s*.*s*. mosquitoes, from a collection originally obtained from Njage Village in Ifakara District of South East Tanzania and maintained in ICIPE insectaries, were used in this study. The insects were reared at ICIPE, Nairobi, Kenya according to the established WHO (1996) protocol. Briefly, the larvae were reared at 32–36 °C and fed on TetraMin^®^, Tetra GmbH, Germany, while the adults were maintained on 6% glucose solution and the females fed on human blood thrice a week. Rearing temperatures and relative humidity in the adult insectaria were 26–28 °C and 70–80%, respectively.

Repellency tests were done following the WHO, 1996 protocol [51, 52]. Briefly, solutions of human odor blend samples in HPLC-grade acetone were uniformly applied on forearms of volunteer human subjects and exposed to 25 starved mosquitoes for 3 minutes.

Evaluation of the repellent activity of the samples was carried out using human volunteers and 5–7 day-old female *An*. *gambiae* that had been starved for 18 h, but previously fed on 6% glucose solution in a 7×5×3 m room maintained at 30–32°C and 65–80% relative humidity. Only volunteers with mild or no allergic reactions to mosquito bites participated in the experiments and were not allowed to apply lotions, perfumes, oils or perfumed/fragrant soaps on the day of the assay. Bioassays of the foot odor constituents at 10^−5^, 10^−4^, 10^−3^, 10^−2^ and 10^−1^ gL^−1^ concentration levels were carried out in aluminium-frame cages (50 × 50 × 50 cm), with aluminium sheet bottom, window screen (mesh size 256) on top and back, clear acrylic (for viewing) on the right and left sides, and a cotton stockinet sleeve for access on the front [52–54]. Test solutions (0.5 mL), in HPLC grade acetone, were dispensed on one of the forearms of a volunteer from the wrist to the elbow. The rest of the hand was covered with a glove. HPLC grade acetone (0.5 mL) was dispensed on the other forearm to serve as control. The control and test arms were interchanged regularly to eliminate any bias. Experimental 5-day-old female *An*. *gambiae* mosquitoes (n ₌ 100) were released into the bioassay cage in paper cups, followed by introduction of control arm into the cage and the insect given 3 min to settle prior to recording the number of mosquitoes landing for blood meal. The mosquitoes that landed on the hand were recorded and then shaken off before imbibing any blood. Subsequently, the test arm was introduced into the cage for the same duration and the number of landing insects recorded. Different samples were tested sequentially starting with the lowest concentration to the highest one [53, 54].

### Ethics statement

The study was approved by the National Ethical Review Committee based at the Kenya Medical Research Institute (KEMRI) and informed written consent was obtained from each of the participants/volunteers before the commencement of experiments involving human subjects. All participants involved in the study were adults and not minors.

## Results and discussion

### Chemical composition of the foot odor

**Fig 2:**
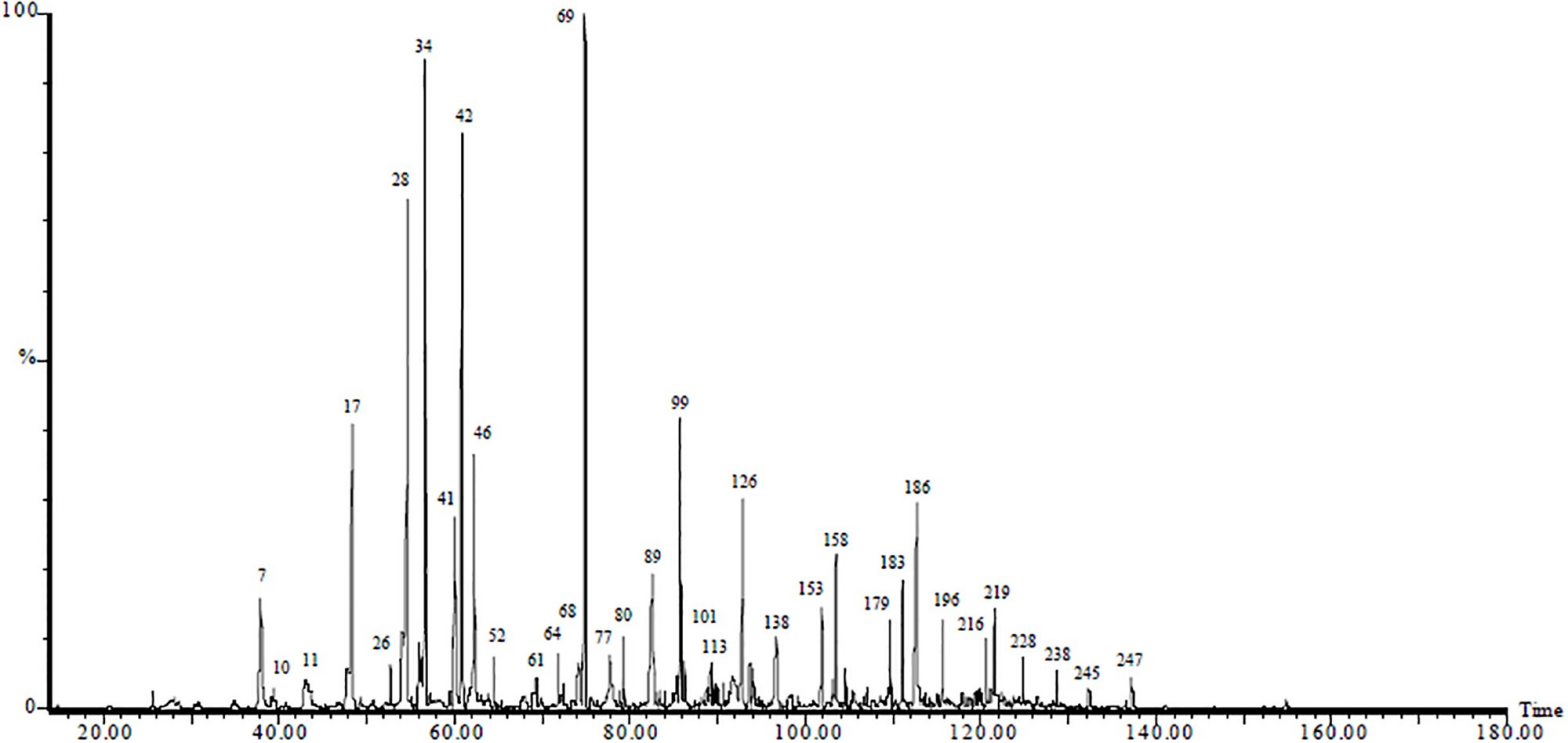
GC profile of most attractive human foot odor. The numbers represent individual peaks in the foot odour as they get eluted from the GC column; Peak 69 is geranylacetone and major component but not bioactive, while peak 10 *n-octanal*, 17 (*n-*decanal), 34 (*n*-decanal), 42 (4-ethylacetophenone), 52 (*n*-undecanal) and 77 (*n*-dodecanal) are bioactive among the other electro-physiologically active ones that are too tiny to be labelled.

The GC chemical profile of the most attractive human foot odor which were found to be qualitatively comparable with those previously reported by Bernier et al. [25, 55] is shown in Fig 2. Interestingly, several compounds: *E*-3-methyl-2-hexenoic acid, *Z*-3-methyl-2-hexenoic acid and 7-octenoic acid [56], propionic acid, butyric acid [38, 44, 57]; 1-octen-3-ol, 4-methylphenol (*p*-cresol), *L*-lactic acid, indole, and 3-methylbutanol [59] that had previously been reported as olfactory stimulants of *An*. *gambiae* [56–59] were not detected in the human foot odor in our study. However, 2-cresol (*o*-cresol) and 3-methylbutamine were identified in the current work instead of 4-cresol (*p*-cresol) and 3-methylbutanol, respectively, which had been previously reported as olfactory stimulants [59]. The absence from foot volatile collections of some compounds previously identified in human skin emissions could be due to variations between body parts, method of collection, or variation between human subjects [22, 26]. Specifically, our inability to detect *L*-lactic acid, an established mosquito olfactory stimulant [60], in human foot odor could be due to static trapping method we used vis a vis its low volatility and/or high polarity leading to its failure to be adsorbed onto and/or desorbed from Porapak Q.

### Electro-antennogram (EAG) assay of the human foot odor volatile components

**Fig 3:**
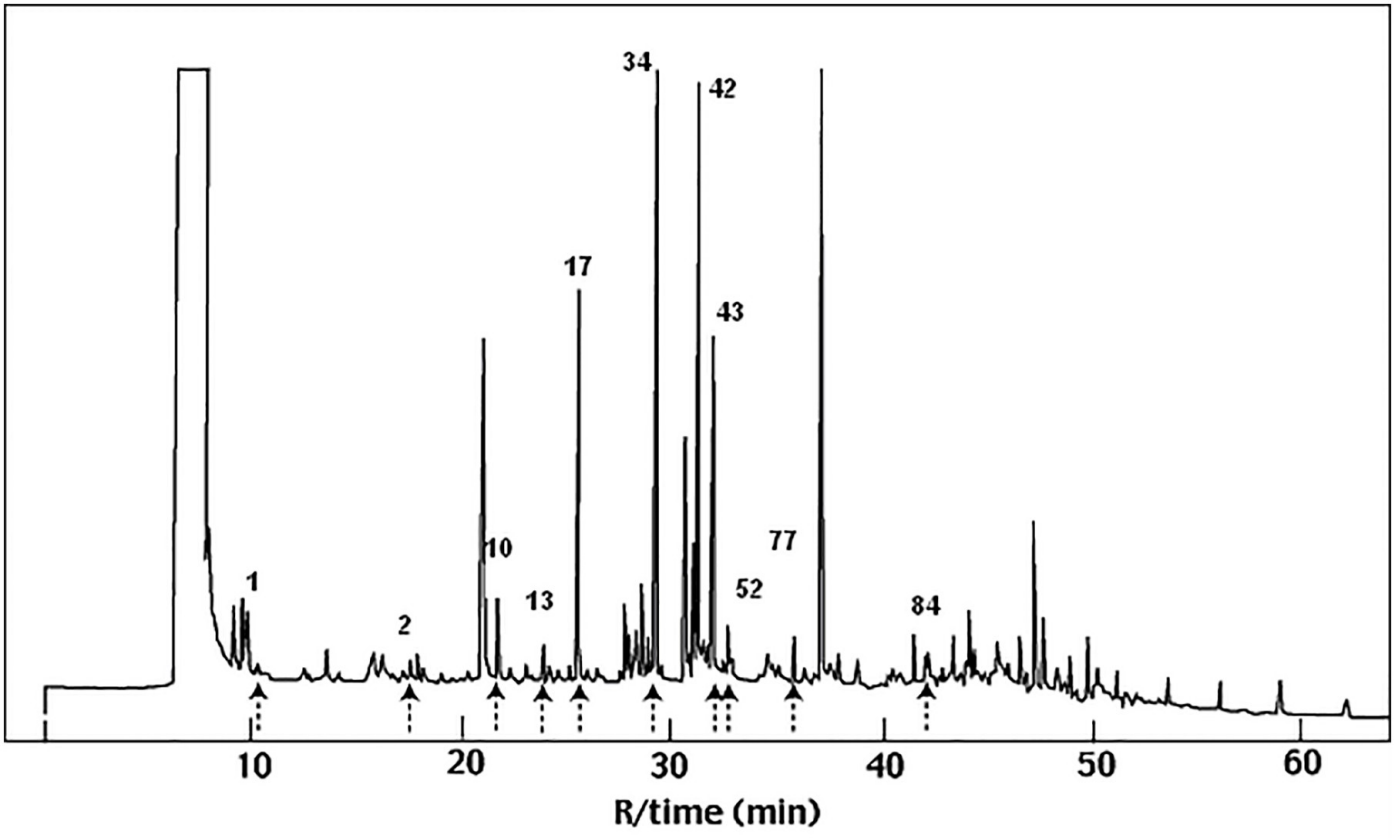
The GC-FID profile with the EAD-active components (numbered) of the most attractive human foot odor.

**Fig 4:**
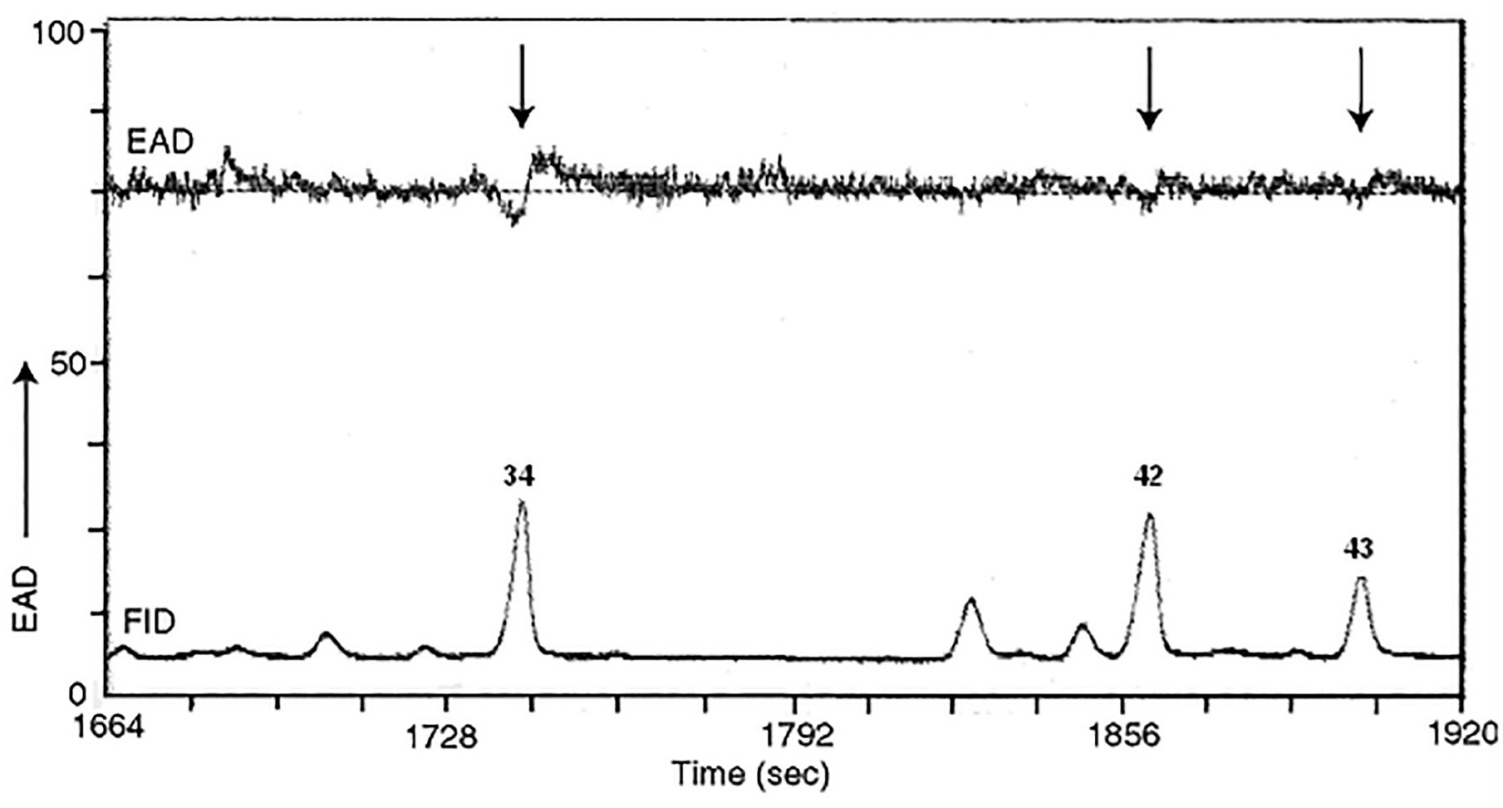
The EAD and FID signals for a section of the foot odor chemical profile.

The GC-EAG analysis of the human foot odor volatiles from the most attractive human subject revealed 11 EAG-active components which have been identified as: *i*-butyric acid (**1**), *i*-valeric acid (**2**), *n*-octanal (**10**), 2-methylphenol, (**13**), *n*-nonanal (**17**), *n*-decanal (**34**), 4-ethylacetophenone (**42**) and 4-ethoxyacetophenone (**43**), *n*-undecanal (**52**), *n*-dodecanal (**77**) and *n*-tridecanal (**84**) (Fig 3). Decanal (**34**) elicited the strongest EAG activity while the rest of the compounds exhibited weak responses (Fig 4).

Detection of volatile semio-chemicals by mosquitoes occurs mainly via the olfactory neurons on the antennae [61]. In earlier studies, *An*. *gambiae s*.*s*. EAG responses to human-specific sweat components: (E)-3-methyl-2-hexenoic acid, (Z)-3-methyl-2-hexenoic acid and 7-octenoic acid were reported to elicit EAG activity using whole antennae [58]. Other known olfactory stimulants of *An*. *gambiae* include: propionic acid, butyric acid, *i*-butyric acid [38, 44, 57], *i*-valeric acid, and *L*-lactic acid; 4-methylphenol (*p*-cresol); 1-octen-3-ol [56, 58] and 3-methyl-1-butanol [59]; heptanal, octanal, and nonanal [45]; geranylacetone and 6-methyl-5-hepten-2-one; and indole [59]. Although the EAG activity of geranylacetone and 6-methyl-5-hepten-2-one have been previously reported for *An*. *gambiae* [59] and *Aedes aegypti* [62], they neither elicited any responses in the current GC-EAG experiment nor exhibited any behavior modifying activity despite being the major components of the human foot odor. Unlike in the previous studies, lack of EAG activity of geranylacetone and 6-methyl-5-hepten-2-one, the major components of human foot odor, may be due to the improvement in GC resolution of all the constituents thus ensuring that no bioactive compounds co-eluted with them.

Of the 11 EAG-active components of the human foot odor identified in this study, four compounds: have been shown to elicit EAG-activity, attract, and or play a synergistic role in the attraction of mosquitoes [44, 45, 56, 63, 64], while the remaining seven compounds are newly identified.

The origin of the short chain carboxylic acids has been established to be due to the metabolism of aliphatic amino acids present in eccrine sweat by the *Staphylococci* [64–66]; and they have the potential to synergize the attractiveness of lactic acid, in the sweat, to *An*. *gambiae* [67].

### Semi-field bioassay of the EAG-active blends of components of human foot odor

**Fig 5:**
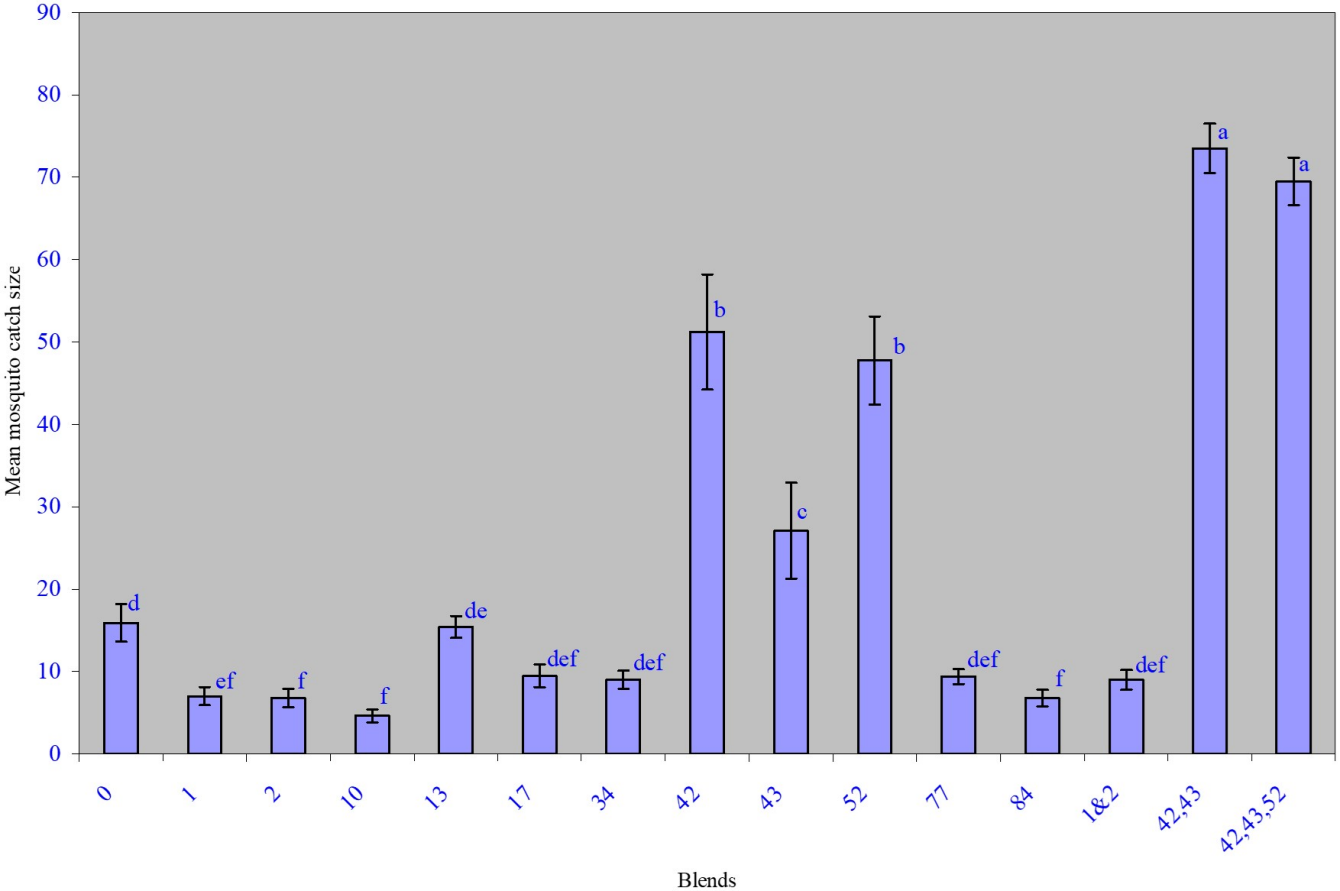
Mean mosquito catch sizes* in CFG traps baited with synthetic blends of EAD active foot odor components. *Based on 8 replicates of mosquito catch sizes per blend; blends not sharing the same letters are significantly different (p = 0.0001).

The blend ratios of the 11 EAD-active human odor constituents used in subtraction bioassays, the missing/excluded components and the mean mosquito catch sizes are summarized in Table 1. The largest drop in activity of the blends occurred in A10, which did not contain *n-*octanal (**10**) (Fig 5). The mean (SNK—test) mosquito catch sizes for blends A1, A2, A10 and A84 were significantly lower (p = 0.0001) than that for blend A0, which had all the 11 EAG active components. However, the exclusion of nonanal (**17**), decanal (**34**), undecanal (**52**) and dodecanal (**77**) individually from the blends A17, A34, A52 and A77, respectively, caused the least significant drop in mean mosquito catch sizes compared to A0. On the other hand omission of 4-ethylacetophenone (**42**) and 4-ethoxyacetophenone (**43**) individually in blends A42 and A43 significantly increased (p = 0.0001) the mean catch sizes of these blends compared to A0; while there was no significant difference between the catch sizes due to A13 and A0. The mean catch sizes due to the blends with the two or three repellent compounds missing (A42, 43 and A42, 43, 52) were significantly higher (p = 0.0001) than the catch sizes obtained with the blends with only one or none of them missing (A42, A43, A52 and A0).

**Table 1:** Compositions of the artificial blends of EAG-active components of human odor and mean mosquito catch sizes.

S/No.	Blend	Omitted Odour Component(s) ^†^	Ratio of Odour Components	Mean Mosquito Catch Size* ± SE
1.	A0	None	4:9:6:1:95:250:256:132:17:15:7	15.875 ± 2.255^d^
2.	A1	*i*-Butryic acid (1)	9:6:1:95:250:256:132:17:15:7	7.000 ± 1.134^ef^
3	A2	*i-*Valeric acid (2)	4:6:1:95:250:256:132:17:15:7	6.750 ±1.146^f^
4.	A10	Octanal (10)	4:9:1:95:250:256:132:17:15:7	4.625 ±0.754^f^
5.	A13	Cresol (13)	4:9:6:95:250:256:132:17:15:7	15.375±1.252^de^
6	A17	Nonanal (17)	4:9:6:1:250:256:132:17:15:7	9.500 ±1.402^def^
7.	A34	Decanal (34)	4:9:6:1:95:256:132:17:15:7	9.000 ±1.982^def^
8.	A42	4-Ethylacetophenone (42)	4:9:6:1:95:250:132:17:15:7	51.250 ±7.015^b^
9.	A43	4-Ethoxyacetophenone (43)	4:9:6:1:95:250:256:17:15:7	27.125 ±5.848^c^
10.	A52	Undecanal (52)	4:9:6:1:95:250:256:132:15:7	9.500 ±1.134^def^
11.	A77	Dodecanal (77)	4:9:6:1:95:250:256:132:17:7	9.375 ±0.981^def^
12.	A84	Tridecanal (84)	4:9:6:1:95:250:256:132:17:15	6.750 ± 3.406^f^
13.	A42,43	4-Ethylacetophenone (42) 4-Ethoxyacetophenone (43)	4:9:6:1:95:250:17:15:7	75.000±2.252^a^
14.	A42,43,52	4-Ethylacetophenone (42) 4-ethoxyacetophenone (43) Undecanal (52)	4:9:6:1:95:250:15:7	73.50 ±2.909^a^

A0—The artificial blend of all EAG-active compounds (*i*-butyric acid, *i*-valeric acid, octanal, cresol, nonanal, decanal, 4-ethylacetophenone, 4-ethoxyacetophenone, undecanal, dodecanal and tridecanal) in human foot odor in the indicated respective ratios.

A1- A84—Artificial blend of all the EAG-active compounds in the respective ratios minus the ones indicated as omitted.

*Based on 8 replicates on number of mosquitoes responding to the tested blend;

^†^Peak number of compound subtracted from the initial blend, A0 (contained all the 11 EAD active compounds);

Means with the same letter are not significantly different (SNK test, p = 0.0001).

*i*-Butyric acid (**1**), *i*-valeric acid (**2**), *n-*octanal (**10**) and *n-*tridecanal (**84**) could be critical/important kairomonal components of the human foot odor as observed in reduce mean mosquito catch size of blends where they are individually missing. On the other hand, nonanal (**17**), decanal (**34**), undecanal (**52**) and dodecanal (**77**) have mild kairomonal activity at low natural concentrations due to the insignificant differences observed on the mean mosquito catch size of the blends without them. The kairomonal activity of the aldehydes seem to be synergistic. On the other hand, 4-ethylacetophenone (**42**), 4-ethoxyacetophenone (**43**) and 2-methylcresol (**13**) have significant repellent effect on *An*. *gambiae* mosquitoes. The repellent activity of the 4-ethylacetophenone (**42**) and 4-ethoxyacetophenone (**43**), was further confirmed in the bioassay of blend A42,43 without the 2 or 3 repellent compounds. The repellent effect of the two acetophenones is additive as evident in the mean mosquito catch sizes for A42 and A43 individually compared to A42, 43 blend.

In high unnatural concentrations, 4-ethoxyacetophenone was found to be the most repellent component of the human foot odor with protective efficacy of 100 ± 0% at the highest dose (10%), compared to 70.53 ± 3.81 and 68.2 ± 4.19% for 4-ethylacetophenone and undecanal, respectively, in normal repellency assays. The C_4_ and C_5_ carboxylic acids and C_8_ to C_13_ straight chain aldehydes have been patented as attractants for blood-feeding insects [68, 69] while the acetophenones and undecanal have also been reported in another patent publication as repellents for blood-feeding insects [70]. 4-Ethoxy acetophenone has previously been reported as a repellant for the honey bee, *Apis mellifera* L in semi-field trials [71]. A related compound 4-methoxyacetophenone has also been patented as a repellent for Scotylid beetles in coniferous plantations [72]. It is also known that acetophenone forms the basic structure of several non-lethal avian repellent compounds [73, 74].

It is interesting to note that while undecanal (**52**) exhibits mosquito repellent activity at high unnatural concentrations as previously observed in direct bioassay of the single compounds in the laboratory [70], it exhibits mild kairomonal activity at low natural concentrations under semi-field conditions as demonstrated by the insignificant effect on the mean catch size for blends A52 and A42,43,52 (Fig 5) caused by its exclusion singly and together with the two acetophenones, respectively.

**Fig 6:**
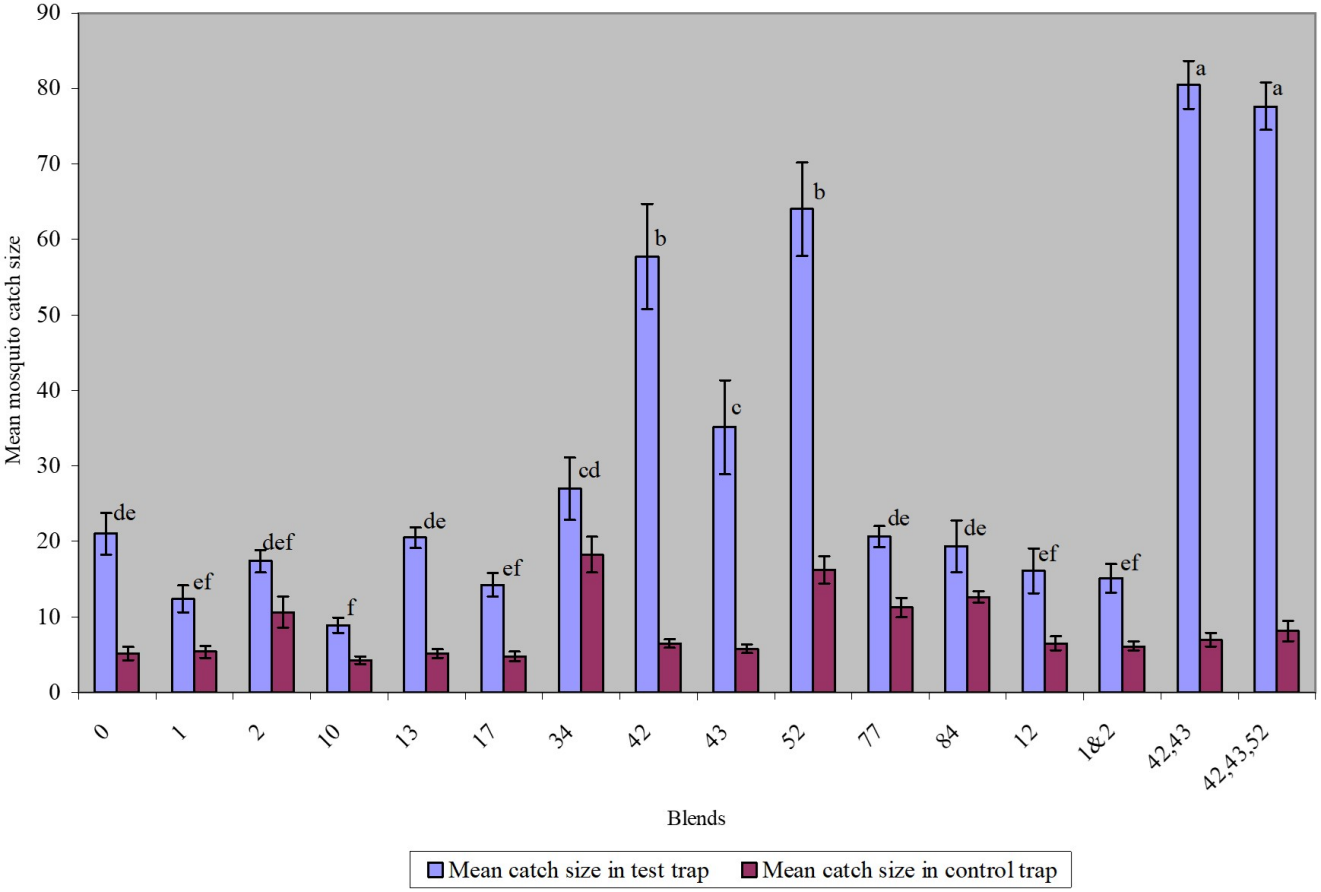
Comparison of mean mosquito catch sizes* in test and control CFG traps. *Based on 8 replicates of mosquito catch sizes per blend; blends not sharing the same letters are significantly different (p = 0.0001).

The semi-field experimental data (Fig 6) confirmed that there was a significant difference in the mean number of mosquitoes caught in traps baited with the most attractive blends compared to the various controls. In all the three cases, the CFG traps baited with the eight-component blend trapped significantly more mosquitoes compared to the other lures. The average mosquito catch for the eight-component blend-baited CFG trap was 129 ± 9.7 compared to 23.5 ± 11.6 for the CDC trap in the experiment, whereas it trapped 105 ± 14.5 mosquitoes compared to 45.5 ± 5.6 for the human bed-net trap; and 35.5 ± 5.7 compared to 19.5 ± 4.2 for CFG trap baited with socks treated with the natural foot odor from most attractive human volunteer.

The use of host-produced infochemicals in manipulating behavioral response of blood-feeding insects is well documented in tsetse fly and tick control among other blood feeding arthropods [47, 75]. Several phenolic compounds including phenol, 3-methylphenol, 2-methoxy-4-methylphenol, 3-ethylphenol, and 4-ethylphenol, 3-propylphenol and 4-propylphenol [76] are known short range testse fly attractants from oxen and buffaloes [48, 75–77] urine. They are produced from pro-attractants consisting of a mixture of glucuronates and sulphates that get broken down due to the microbial activity of various bacteria to the corresponding phenolics [76]. Similarly, animal hosts of disease vectors also produce volatiles that protect them from attacks and threats of their enemies. For instance, 3-cresol, guaiacol, geranylacetone, pentanoic acid and δ-octalactone, have been reported to be tsetse fly repellents that are produced by the waterbuck (a non-preferred host) and protect it from attack by the tsetse flies [48, 78–80]. Waterbuck allomones have been shown to have the potential of protecting oxen from tsetse flies [80].

## Conclusion

The C_4_ and C_5_ carboxylic acids and C_8_ to C_13_ straight chain aldehydes are attractants for *An*. *gambiae* at low natural concentrations while the two acetophenones are repellents. Undecanal is an attractant at low concentrations but a repellent at high unnatural concentrations. The attractants are ineffective when presented individually to mosquitoes, but are active as a blend implying that they act synergistically while the two repellent acetophenones act additively. This work underscores the relevance of subtraction/exclusion assays in conferring activity to the blended components of the human foot odor, additive, synergistic and collective activity of the *An*. *gambiae* olfactory stimulants. The kairomonal blends identified from the foot odor together with known mosquito repellents could be used to reduce host-vector contact in a pull–push control strategy. Moreover, the attractive blend could be exploited to lure the vectors into traps, where they can die from hunger and/or desiccation. Alternatively, the trapped insects may be sterilized, or subjected to entomo-pathogens (bacteria, fungi, viruses, and nematodes—like *Bacillus thurigensis*, *Bauvaria bassianna* among others) or parasitoids before being released into the wild for population control. Furthermore, they may also be useful in routine mosquito population monitoring to forecast disease epidemics and dictate timely interventions such as insecticide application and anti-malarial drug procurement.

## Supporting information

S1 Plate Adsorbent sachets on the feet of the human subject. (DOCX)
