# Supplementary figures and images for: Semiochemical signatures associated with differential attraction of Anopheles gambiae to human feet
Source: PLoS One. 2021 Dec 3;16(12):e0260149. doi: 10.1371/journal.pone.0260149 (PMC8641859; doi:10.1371/journal.pone.0260149)

Plate 1: Adsorbent sachets on the feet of the human subject


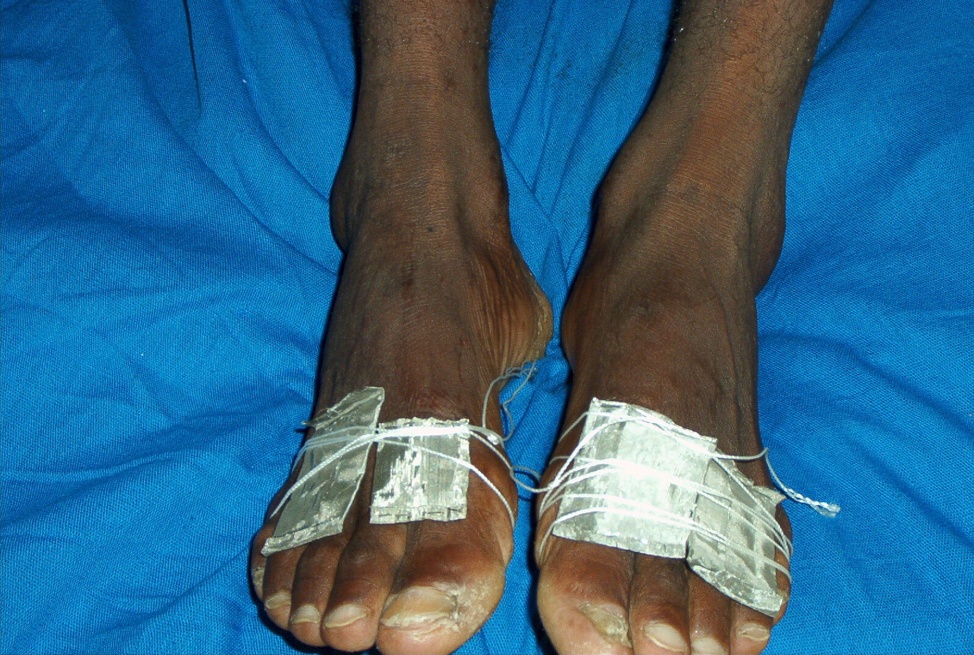

Supplement: S1 Plate — (DOCX) [file pone.0260149.s001.docx]
